# Hemodynamic Relevance of Anomalous Coronary Arteries Originating From the Opposite Sinus of Valsalva-In Search of the Evidence

**DOI:** 10.3389/fcvm.2020.591326

**Published:** 2021-01-21

**Authors:** Marius Reto Bigler, Afreed Ashraf, Christian Seiler, Fabien Praz, Yasushi Ueki, Stephan Windecker, Alexander Kadner, Lorenz Räber, Christoph Gräni

**Affiliations:** ^1^Department of Cardiology, Inselspital, Bern University Hospital, University of Bern, Bern, Switzerland; ^2^Department of Cardiovascular Surgery, Centre for Congenital Heart Disease, Inselspital, Bern University Hospital, University of Bern, Bern, Switzerland

**Keywords:** anomalous coronary arteries originating from the opposite sinus of Valsalva (ACAOS), multimodality imaging, hemodynamic relevance, fixed vs. dynamic stenosis, anomalous aortic origin of the coronary artery (AAOCA), dobutamine-volume challenge, fractional flow reserve (FFR), intravascular ultrasound (IVUS)

## Abstract

Coronary artery anomalies (CAA) represent a heterogeneous group of congenital disorders of the arterial coronary circulation, defined by an anomalous origin of the coronary ostium and/or vessel course. Of particular interest are anomalous coronary arteries originating from the opposite sinus of Valsalva (ACAOS). The interarterial variants (with the anomalous vessel situated between the great arteries) are historically called “malignant,” based on an anticipated higher risk for myocardial ischemia and sudden cardiac death (SCD), especially affecting young patients during strenuous physical activity. However, the interarterial course itself may not be the predominant cause of ischemia, but rather represents a surrogate for other ischemia-associated anatomical high-risk features. As the exact pathophysiology of ACAOS is not well-understood, there is a lack of evidence-based guidelines addressing optimal diagnostic work-up, downstream testing, sports counseling, and therapeutic options in patients with ACAOS. Therefore, treating physicians are often left with uncertainty regarding the clinical management of affected patients. This review focuses on the pathophysiologic consequences of ACAOS on myocardial ischemia and discusses the concept of the interplay between fixed and dynamic coronary stenosis. Further, we discuss the advantages and limitations of the different diagnostic modalities and give an outlook by highlighting the gaps of knowledge in the assessment of such anomalies.

## Introduction

Coronary artery anomalies (CAA) represent a heterogeneous group of congenital disorders of the arterial coronary circulation, hallmarked by the anomalous origin of the coronary ostium, vessel course, and/or unusual number (1). CAAs are the consequence of an anomalous ingrowth from the initially preformed subepicardial vascular plexus into the aortic root during the embryonic period (2, 3). Reflecting this heterogeneity, clinical presentation varies ranging from normal variants [e.g., myocardial bridges, separate origin of the left anterior descending and circumflex artery (4)], which remains often undetected, to potentially life-threatening anomalies (e.g., ectopic origin of a coronary artery from the pulmonary artery). Of particular interest are anomalous coronary arteries with the origin of the anomalous vessel from the opposite sinus of Valsalva (ACAOS), especially if they follow an interarterial course between the great arteries (i.e., aorta and pulmonary artery). This rare congenital abnormality has a prevalence of 0.26% in the general population (0.03% for left coronary ACAOS; L-ACAOS, 0.23% for right coronary ACAOS; R-ACAOS) (5, 6). These interarterial variants are historically referred to as “malignant” based on the anticipated higher risk for myocardial ischemia and sudden cardiac death (SCD), especially affecting young adults during strenuous physical activity (7–11). Indeed, autopsy series showed that ACAOS were in up to one-third the underlying cause of sports-related SCD in young military recruits in the United States (L-ACAOS more frequently than R-ACAOS) (9, 12, 13). However, this proportion does not reflect the absolute risk of SCD in people *living* with ACAOS (14) that remains very low (15). Furthermore, the interarterial course itself may not be the predominant cause of ischemia, but rather represents a surrogate for other ischemia-associated anatomical high-risk features. Nevertheless, the few available professional guidelines recommend strict sports abstinence in patients with interarterial courses (AHA/ACC 2015, Class IIIB/C, ESC 2020 IIIC) and a low threshold for surgical coronary revascularization (ACC/AHA 2008, Class IB/IIa C, AHA/ACC 2015, Class IB, AHA/ACC 2018, Class IB/IIa C, ESC 2020 IC/IIa C) (16–20). There, surgical revascularization demonstrates favorable outcomes, although long-term implication remains unknown (21). As the level of evidence supporting the guidelines about optimal diagnostic work-up, downstream testing, sports counseling, and therapeutic options in patients with ACAOS is limited, treating physicians are often uncertain how they should counsel their patients (22).

Beside young athletes, substantial interest has emerged for the management of older patients with newly diagnosed ACAOS. This is of particular interest, as with the growing use of non-invasive imaging for the evaluation of coronary artery disease (CAD), the number of newly detected ACAOS is growing. Management strategies in the middle-aged and elderly group is even less well-established compared to young individuals, and range from strict sports restriction and/or revascularization to watchful waiting (14, 16–18, 23) (see Table 1 for a summary of available recommendations). The latter strategy (i.e., watchful waiting) is supported by growing evidence for possibly decreasing hemodynamic relevance of the ACAOS above a certain age (24), when symptomatic CAD becomes more prevalent (25). Still, whether older individuals might suffer from a lower ACAOS-related ischemic risk compared to younger individuals (25, 26) remains under debate. Furthermore, as the exact pathophysiology is not completely understood, functional imaging methods routinely used for CAD-evaluation are possibly not directly applicable to rule out ACAOS related hemodynamic relevance.

**Table 1:** Guideline recommendations regarding diagnostic evaluation and treatment in patients with ACAOS.

**ACC/AHA 2008 guidelines for the management of adults with congenital heart disease**	**AHA/ACC 2018 guidelines for the management of adults with congenital heart disease**	**2016 AATS expert consensus guidelines: anomalous coronary artery**	**2015 AHA/ACC scientific statement for competitive athletes with cardiovascular abnormalities**	**2020 ESC guidelines for the management of adult congenital heart disease**	**2020 ESC guidelines on sports cardiology and exercise in patients with cardio-vascular disease**
The evaluation of individuals who have survived unexplained aborted sudden cardiac death or with unexplained life-threatening arrhythmia, coronary ischemic symptoms, or LV dysfunction should include assessment of coronary artery origins and course. (I B)	Coronary angiography, using ICA, CCTA, or CMR, is recommended for evaluation of ACAOS (I C)	Individuals with suspected ACAOS should undergo TTE to identify the origin and course of the proximal coronary arteries. (I B)	Athletes with R-ACAOS should be evaluated by an exercise stress test. For those without either symptoms or a positive exercise stress test, permission to compete can be considered after adequate counseling of the athlete, taking into consideration the uncertainty of a negative stress test (IIa C)	Non-pharmacological functional imaging (e.g., nuclear study, echocardiography, or CMR with physical stress) is recommended in patients with coronary anomalies to confirm/exclude myocardial ischemia (I C)	When considering sports activities, evaluation with imaging tests to identify high-risk patterns and an exercise stress test to check for ischaemia should be considered in individuals with ACAOS. (IIa C)
CT or CMR angiography is useful as the initial screening method in centers with expertise in such imaging (I B)	Anatomic and physiological evaluation should be performed in patients with ACAOS (I C)	Additional imaging studies, such as CCTA or CMR are reasonable to better visualize the coronary artery anatomy and to confirm the diagnosis. (IIa B)	Athletes with an L-ACAOS should be restricted from participation in all competitive sports before surgical repair (independent from symptoms) (III B)	Surgery is recommended for ACAOS in patients with typical angina symptoms who present with evidence of stress-induced myocardial ischemia in a matching territory or high-risk anatomy (I C)	In asymptomatic individuals with a CAA without anatomical high-risk features, competition may be considered, after adequate counseling on the risks, provided there is absence of inducible ischaemia. (IIb C)
Surgical coronary revascularization should be performed in patients with L-ACAOS with/without documented ischemia R-ACAOS with documented ischemia (I B)	Surgery is recommended for ACAOS (L-and R) for symptoms or diagnostic evidence consistent with coronary ischemia attributable to the ACAOS (I B)	In asymptomatic patients without a history of aborted SCD, exercise stress testing combined with nuclear perfusion scan or echocardiographic imaging should be used to assess the potential ischemic burden of ACAOS (I B)	Non-operated athletes with a R-ACAOS who exhibit symptoms, arrhythmias, or signs of ischemia on exercise stress test should be restricted from participation in all competitive sports (III C)	Surgery should be considered in asymptomatic patients with ACAOS and evidence of myocardial ischemia (IIa C)	After surgical repair of an ACAOS, participation in all sports may be considered, at the earliest 3 months after surgery, if they are asymptomatic and there is no evidence of inducible myocardial ischaemia or complex cardiac arrhythmias during maximal exercise stress test. (IIb C)
Surgical coronary revascularization can be beneficial in the setting of documented vascular wall hypoplasia, coronary compression, or documented obstruction to coronary flow, regardless of inability to document coronary ischemia (IIa C)	Surgery is reasonable for L-ACAOS in the absence of symptoms or ischemia (IIa C)	ICA should be performed in suspected ACAOS if the anatomy cannot be defined with non-invasive imaging, and in adults with risk factors for coexistent atherosclerotic CAD (I B)		Surgery should be considered in asymptomatic patients with L-ACAOS and no evidence of myocardial ischemia but a high-risk anatomy (IIa C)	Participation in most competitive sports with a moderate and high cardiovascular demand among individuals with AOCA with an acutely angled take-off or an anomalous course between the large vessels is not recommended. (III C)
Delineation of potential mechanisms of flow restriction via IVUS can be beneficial in patients with ACAOS (IIa C)	Surgery for ACAOS is reasonable in the setting of ventricular arrhythmias (IIa C)			Surgery may be considered for symptomatic patients with ACAOS even if there is no evidence of myocardial ischemia or high-risk anatomy (IIb C)	
	Surgery or continued observation may be reasonable for asymptomatic patients with ACAOS without ischemia or anatomic or physiologic evaluation suggesting potential for compromise of coronary perfusion (IIb B)			Surgery may be considered for asymptomatic patients with L-ACAOS without myocardial ischemia or high-risk anatomy when thy present at young age (<35 years) (IIb C)	

*ACAOS, anomalous coronary arteries with the origin of the anomalous vessel from the opposite sinus of Valsalva; CAD, coronary artery disease; CCTA, coronary computed tomography angiography; CMR, cardiac magnetic resonance; ICA, invasive coronary angiography; IVUS, intravascular ultrasound; SCD, sudden cardiac death*.

In this review, we will focus on the pathophysiologic consequences of ACAOS on myocardial ischemia. In addition, we will discuss the concept of the interplay of fixed and dynamic stenosis in ACAOS, which is important toward optimal stress test modality selection. Finally, we will discuss advantages and limitations of the different diagnostic modalities and provide an outlook by highlighting the gaps of knowledge in the evaluation of ACAOS patients.

## Methods

The initial literature research started systematically on Medline Ovid and Pubmed with focus on peer-reviewed, English publication on coronary anomalies, diagnostic modalities, and myocardial ischemia within the last 20 years (i.e., 2000–2020). This resulted in 588, respectively, 518 articles, which we further decreased to 201 full text analysis. These were initially included and read by MRB and/or AA. However, as old autoptic studies as well as echocardiography papers were missing, we manually search for the most referenced papers within this topic. Thus, the presented review is methodological narrative.

## Pathomechanisms of Ischemia in ACAOS

**Figure 1:**
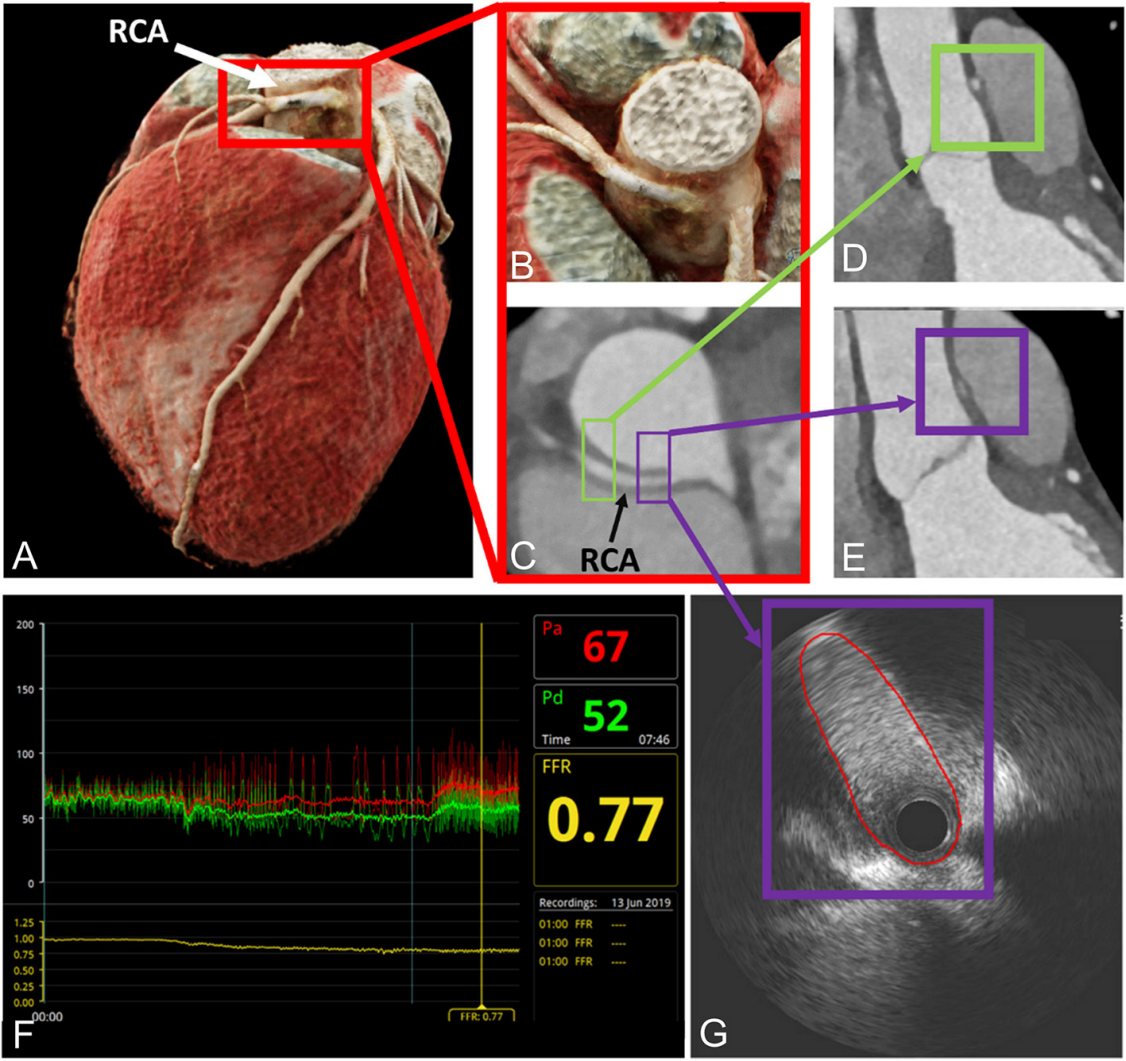
Depiction of anatomical high-risk features in a patient with right ACAOS by coronary computed tomography angiography and invasive coronary angiography. **(A–C)** Illustration of the interarterial course by CCTA; **(D,E)** depiction of anatomical high-risk features [acute-take off-angle, proximal narrowing and oval vessel shape; purple box proximal segment, also called Angelini/Cheong sign (29), green box distal segment]; **(F,G)** invasive assessment with a positive FFR and demonstration of lateral compression by intravascular ultrasound. Red line depicts lumen contour. With permission from Elsevier. Bigler et al. (30).

Although there have been several attempts to uncover the pathophysiology of ACAOS during the previous decades, the underlying mechanisms of ischemia remain ambiguous. Historically, the interarterial course was thought to be the crucial abnormality assuming a scissor-like mechanism created by the close proximity of the aorta and pulmonary artery, especially during exertion (7). Considering the pressure condition in the respective circulatory systems, it is unlikely that the low-pressure pulmonary artery would develop substantial counterforce to occlude the anomalous coronary artery. Furthermore, at the site of closest aortopulmonary proximity, the anomalous segment usually runs inside the aortic wall (8, 27, 28). Therefore, the interarterial course may act only as a surrogate for other anatomical high-risk features like slit-like ostium, acute take-off angle, proximal narrowing (also referred to as hypoplasia) with elliptic vessel shape and intramural course (i.e., course within the tunica media of the aortic wall in; see Figure 1) (1, 24, 28, 31–39). Consequently, terminology should focus on these features rather than the interarterial course. Beside these anatomic features supported by a large body of evidence, other postulated mechanisms are dynamic lateral compression of the intramural segment (7, 27), flap-like closure of the narrowed ostium (24, 40), and increased vulnerability to coronary spasm (41). However, coronary spasms are rarely observed in clinical practice, unless catheter cannulation inadvertently results in trauma (41, 42). Especially in ACAOS with intramural course, coronary spasm appears implausible because of the embedment of the ACAOS within the aortic tunica media, a layer of elastic tissue without functional smooth muscle cells (43). In addition, provocative testing for coronary spasm using ergonovine elicits no spasticity of the ectopic segment suggesting that spasm is not contributing to ischemia in ACAOS (27, 44). Similarly, the flap-like closure mechanism is not observed in clinical practice and has only been reported in autoptic studies (24, 40). The failure of demonstrating these mechanisms *in vivo* may be due to the dynamic nature of the phenomenon, which may be missed by imaging. Alternatively, reproducibility may be limited owing to technical issues [e.g., inadequate spatial resolution of non-invasive imaging or blockade of the flap by the intravascular ultrasound (IVUS) or optical coherence tomography (OCT) probe during invasive assessment].

The anatomic high-risk feature of a slit-like ostium at the ectopic origin is defined as a ≥50% reduction of the minimal lumen diameter compared to the normal distal reference diameter (36) [ <50% = oval ostium (36)] and best corresponds to the concept of relevant coronary stenosis known from CAD. Thus, the deformed coronary ostium with a decreased cross-sectional area acts as an ostial stenosis. In a small study, Kaushal et al. compared the mean ostial diameter of anomalous coronaries to those of normal vessels in 27 young patients undergoing surgical correction of ACAOS and found a significant caliber difference (mean diameter 1.5 ± 0.4 mm vs. 3.3 ± 0.8 mm) (45). Accordingly, narrowing of the proximal segment reduces the cross-sectional area in the interarterial part, the relevance of which can be measured using percent diameter stenosis of the anomalous in relation to the unobstructed, distal segment [i.e., (reference area—stenosis area)/reference area^*^100] (46, 47). In case of a stenosis above 50%, revascularization of the proximal vessel may be considered in symptomatic older patients with R-ACAOS (46, 48). Of note and similar to atherosclerotic lesions, not only percent diameter stenosis but also its length affects the hemodynamic relevance directly.

An acute take-off angle (below 45°), defined as an axial course of the proximal segment tangential to the great vessel circumference (40, 49), was previously associated with symptoms (36, 47). Furthermore, kinking of the anomalous coronary artery during exercise, i.e., decrease of the acute take-off angle and consequently increased narrowing at the ostium, was proposed as a contributing ischemia-inducing mechanism (38, 50).

**Figure 2:**
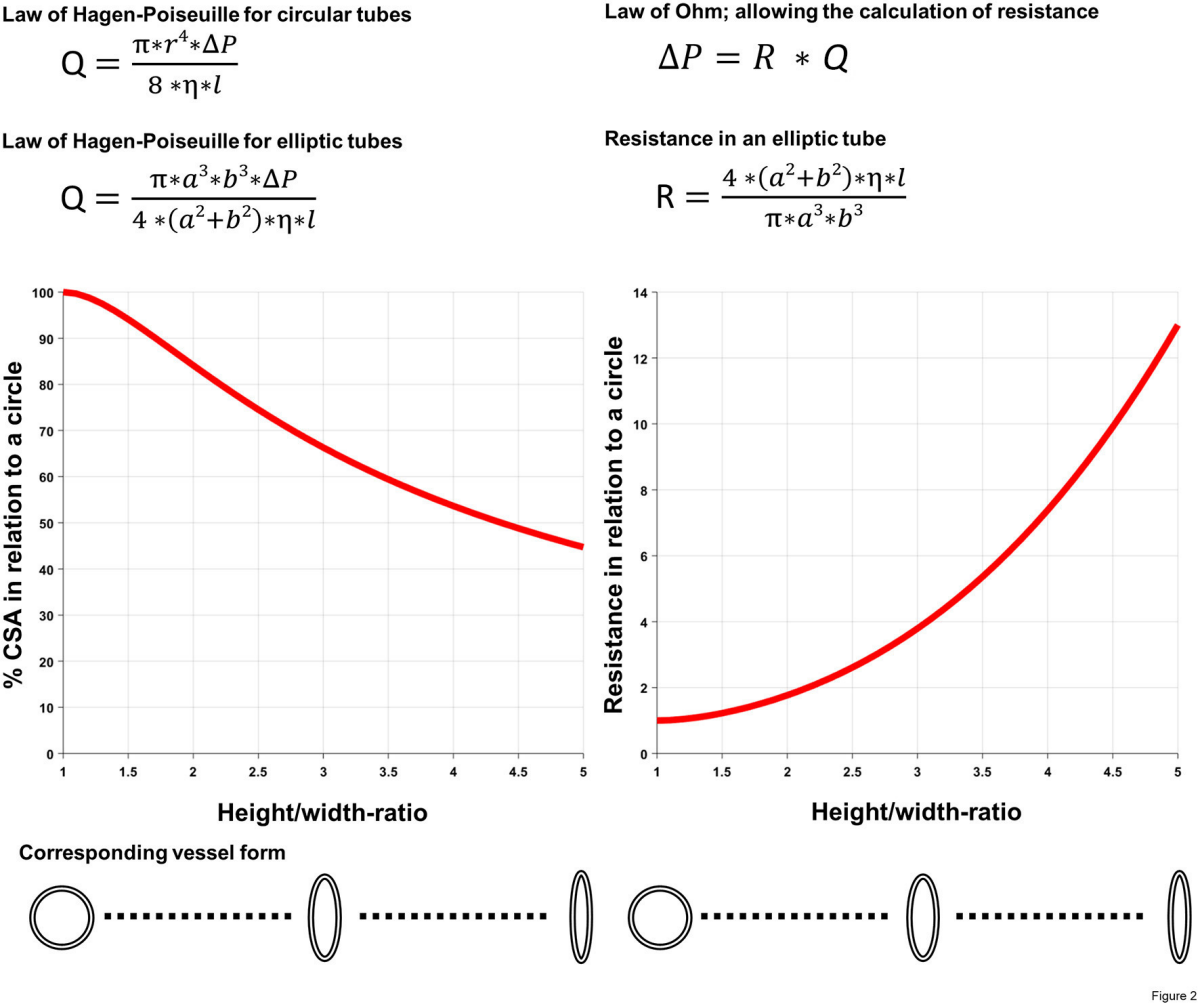
Illustration of the impact of deformed vascular shape (i.e., lateral compression). Using two basic equations of fluid dynamics (law of Hagen-Poiseuille and law of Ohm) as well as common geometric formulas for the calculation of the cross-sectional area (CSA) in different forms, decreasing cross-sectional area, respectively, the increasing resistance as a function of the height/width ratio (i.e., a/b-ratio) in a vessel with a given (i.e., fixed) circumference was calculated. Q, volumetric flow rate; r, radius of the pipe; ΔP, pressure gradient; η, dynamic viscosity; l, length of the pipe; a, short semi-axis of the elliptic pipe, b, long semi-axis of the elliptic pipe; R, resistance; Equations of fluid dynamics taken from (60).

Finally, the intramural course is probably the most threatening feature in terms of hemodynamic relevance (10, 51). As shown by several studies, the length of the intramural segment is associated with an increased risk for ischemia (28, 36, 45, 52). In addition, an elliptic proximal vessel shape [defined as height/width ratio of >1.3 (53)] is frequent within the intramural segment, and the deformation [also called lateral compression, dependent from the cardiac phase, i.e., more pronounced during systole than diastole (54)] has been shown to increase during physical activity with augmented great vessel wall stress (27, 54–59). Taken into account the law of LaPlace [wall stress = (transmural pressure ^*^ radius)/(2 ^*^ wall thickness)], the augmented wall stress affects in particular the intramural segment, where there is a substantial decrease in aortic wall thickness. The latter phenomenon is additionally exacerbated by the increasing artery diameter during physical exercise, thereby producing a lateral compression sufficient to cause myocardial ischemia even during diastole. This anatomic feature is not only relevant due to the reduced cross-sectional area compared with a round vessel shape, but also due to higher resistance as shown by the underlying mechanics, i.e., the law of Hagen-Poiseuille (60). Figure 2 demonstrates the decreasing cross-sectional area and the increasing resistance, respectively, as a function of the height/width ratio in a vessel with a given circumference. Furthermore, as outlined by the position as a denominator in the applied law, intramural length directly increases resistance to flow as well (60). Figure 2 is a theoretical model of the effect of vessel deformation with the limitation that deformation will rarely result in a perfect elliptic shape. Nonetheless, it demonstrates the increasing resistance along the anomalous segment during progressive deformation (which would be even higher with irregular deformation and consecutive turbulent flow) and the need for compensatory coronary vasodilatation for the preservation of adequate perfusion at the expense of decreased coronary flow reserve (CFR). This effect was illustrated in a case report by Brandt et al., where the authors measured CFR during surgical revascularization and demonstrated a decreased CFR when the periphery was supplied by the native vessel compared to the graft (44).

### Two-Tier Concept

Combining the features outlined above, we support a two-tier concept for the pathomechanisms of ischemia in ACAOS (41). In this concept, the occurrence of ischemia is based on the extent of a fixed (anatomic high-risk features of slit-like ostium and proximal narrowing) and a dynamic (acute take-off angle, intramural course with the elliptic vessel shape) component. In previous studies, none of the anatomic features taken separately correlated with ischemia, indicating a complex interplay between the different components (38, 54). In addition, the hemodynamic relevance depends directly on the supplied viable myocardial mass downstream of the stenosis. Thus, providing an explication for the diverging prognosis of R- and L-ACAOS (9).

Last, ischemia is unlikely to occur every time the patient exercises (61), which suggests the presence of additional factors, e.g., volume status and type of physical activity [isotonic, e.g., cycling or running vs. isometric, e.g., weight-lifting (62, 63)]. Although more SCDs are known in patients participating in dynamic sports, the relevance of this differentiation has yet to be determined.

### Fixed Component

As discussed above, slit-like ostium and proximal narrowing are present at rest and behave in a similar manner to classic coronary lesions. The reduction of the cross-sectional area creates flow restrictions, which can be evaluated by coronary angiography or intravascular imaging (64) and/or the pressure gradient over the stenotic segment (65). Fractional flow reserve [FFR, defined as the hyperemic mean distal coronary artery pressure divided by the simultaneous recorded mean aortic pressure (65)] with hyperemia induced by pharmacological vasodilatation (i.e., adenosine) was used to assess the hemodynamic relevance of ACAOS in multiple studies (56, 66, 67). Interestingly, only a poor correlation with symptoms and/or anatomic features could be documented (66). In fact, these studies assessed the fixed component alone and thus, found no hemodynamically relevant FFR according to the used threshold for atherosclerotic lesions of 0.80 (68). These findings are consistent with the postulated pathophysiology and explained by the fact that the dynamic component cannot be sufficiently reproduced using pharmacological stress with vasodilators.

### Dynamic Component

Anatomic features like acute take-off angle or lateral compression in the intramural segment gain hemodynamic relevance during exercise. With increased heart rate, systolic blood pressure and myocardial contractility, systolic expansion and higher wall stress of the proximal aorta can be observed because of increased dP/dt and stroke volume (69). Thus, lateral compression of the intramural segment and subsequent flow resistance increase as a function of cardiac output and systolic blood pressure, affecting CFR during conditions of increased myocardial oxygen demand. This phenomenon causes myocardial ischemia that cannot be triggered by vasodilatatory drugs. Hence, assessment of the hemodynamic relevance of ACAOS should be performed preferably using physical exercise or dobutamine, a beta2-sympatomimetica that increases heart rate and stroke volume (46, 54, 56). In a study by Angelini et al. (46), intravascular ultrasound (IVUS) during dobutamine infusion directly demonstrated increased lateral compression. Furthermore, Lee et al. (56) conducted a study in 37 patients, where FFR_Dobutamine_ was performed in case of a negative FFR_Adenosine_ showing discrepancies in three patients, as evidence for a dynamic component of ischemia. Of note, in multiple studies, FFR_Dobutamine_ was usually lower or equal to FFR_Adenosine_, revealing the inconstant presence of the dynamic component (56, 67). It is conceivable, that with increasing age, thickening and stiffness of the aortic wall decrease distensibility (69) and thus, the dynamic component loses its relevance. These findings are in line with the autoptic studies by Taylor et al. (24, 38), which reported a decreased risk for SCD beyond the age of 30. However, the simultaneously increasing risk for concomitant CAD may incur myocardial ischemia owing to the development of atherosclerotic lesions (34), which rarely directly affects the anomalous segment (56, 58, 59). However, both factors may potentiate themselves and result in myocardial ischemia.

Besides anatomic high-risk features and concomitant CAD, a recent study demonstrated the hemodynamic relevance of a so far “benign” ACAOS variant with intraseptal course. There, up to 50% of these anomalies showed inducible myocardial hypoperfusion during non-invasive stress testing, which was later confirmed by positive invasive FFR (70). Hence, repeated in-depth hemodynamic evaluation with up-to-date non-invasive and invasive testing will be required to understand the subclassification of ACAOS.

### Substrate for Arrhythmia

Up to 66% of diagnosed ACAOS patients do not report any symptoms (5) and the initial presentation may be sudden cardiac death (71). Thus, diagnostic evaluation should not only obtain evidence for ischemia (which can in turn induce arrhythmia), but also assess possible underlying arrhythmogenic myocardial fibrosis and scar. The latter is suspected to occur in ACAOS as an expression of recurrent minor myocardial ischemia that may serve as the substrate for ventricular tachyarrhythmias (8, 67, 69, 72). Autopsy series demonstrated myocardial fibrosis in a significant number of patients with ACAOS (8). However, the amount of fibrosis that should be considered critical is unknown, as well as the best technique to image such lesions. The management of these patients remains difficult, as it is doubtful whether those with ACAOS and myocardial fibrosis are safe to return to competitive sports after revascularization of the anomaly.

## Diagnostic Modalities

Considering the complex pathomechanisms of myocardial ischemia in patients with ACAOS, the optimal diagnostic modality is not only expected to detect the presence of ACAOS with high accuracy but also to collect additional information on anatomical high-risk features, ischemia, evidence for possible myocardial fibrosis/scar as substrate for ventricular tachyarrhythmias (8) and concomitant CAD (34). Thus, multimodality imaging is necessary to cover this broad range of diagnostic entitlements (5, 22). Table 2 provides an overview of the common methods.

**Table 2:** Overview of the diagnostic modalities for the assessment of ACAOS.

	**Echocardiography**	**CCTA**	**CMR**	**ICA with IVUS/FFR**	**SPECT**	**PET**
**Physical characteristics**						
Spatial resolution	++	+++	++	++++	+	+
Temporal resolution	++/+++^*^	++	++	+++	+	+
**Anatomy of coronary arteries**						
Proximal	+++	++++	++++	+++	-	-
Distal	++	++++	++	+++	-	-
Assessment of vascular territories	-	+++	++	+	-	-
**Anatomic high-risk features in ACAOS**						
Interarterial course	++	++++	++++	++	-	-
*Fixed components*
Slit-like ostium	+	++++	++	+++	-	-
Proximal narrowing	++	+++	++	++++	-	-
*Dynamic components*
Take-off angle	++	++++	++++	+	-	-
Elliptic shape	++	+++	++	++++	-	-
Intramural course	++	++++	+++	++++	-	-
**Physiologic high-risk consequences in ACAOS**						
Ischemia	++^°^	+^**^	++++	+++++	+++^°^	++++^°^
Scar	+	++	++++	-	+++	+++
**Features in patients** ** <30 years**						
Feasibility in children	++++	++	+++	+	++	++
Other concomitant congenital malformations	+++	-	++++	-	-	-
**Features in patients** **>30 years**						
Evaluation of CAD	-	+++	-	++++	-	-
Cardiac function	+++	(+)	++++	++	+	++
**Procedural circumstances**						
Ionizing radiation exposure	-	+	-	++	+++	+++
Required expertise	++++	++	+++	+++++	+++	+++
Duration	++	+	+++	++++	++	++

* *with transesophageal echocardiography*;

** *with CT FFR or possibly CT stress perfusion*;

° *physical exercise possible*.

*CAD, coronary artery disease; CCTA, coronary computed tomography angiography; CMR, cardiac magnetic resonance; ICA, invasive coronary angiography; IVUS, intravascular ultrasound; FFR, fractional flow reserve; SPECT, single-photon emission computed tomography; PET, positron emission tomography*.

*Please note, highest level of evidence for the guideline recommendation is “I B” for all diagnostic modalities (14, 16–18)*.

*Adapted from Gräni C. et al. (22)*.

### Electrocardiogram

The standard 12-lead electrocardiogram (ECG) is a valuable diagnostic modality and important part in daily clinical workup. However, it does not play a role suspect or recognize ACAOS (73). As shown in several reports, resting ECG, even in symptomatic patients, does not show any typical alternations (8, 74–76). Similarly, stress ECG, which has already a limited diagnostic accuracy for the diagnosis of CAD [sensitivity 68%, specificity 77% (77)], is not reliable for the detection of ACAOS-dependent myocardial ischemia (8, 71, 75, 78, 79). If stress ECG may play a role by reproducing symptoms or arrhythmia is unclear (80).

### Echocardiography

Using transthoracic echocardiography (TTE), the origin and the proximal course of the coronary arteries can be assessed non-invasively without radiation exposure (81, 82). Usually, diagnosis by TTE is made from a short-axis view in the plane of the aortic root including focused color Doppler interrogation of the aortic wall to identify an intramural course (52, 83). Furthermore, TTE allows the assessment of ventricular and valvular function as well as evaluation of concomitant congenital heart defects. Taking into account the general good acoustic window in children, TTE is an optimal diagnostic modality for an initial evaluation in a pediatric population, in whom radiation exposure is an issue (14, 61, 76, 84, 85). However, important limitations of TTE are the decreased diagnostic value in adults or patients with limited acoustic window (86) as well as the required experience, resulting in a substantial interobserver variability. This variability was demonstrated in a multicenter study where agreement between the echocardiographic core laboratory and the participating sites was poor (87). For the identification of anatomic high-risk features, higher resolution transesophageal echocardiography (TEE) is needed (81). Functional relevance of ACAOS can be assessed by TTE using either a physical or a pharmacological (usually dobutamine) stress looking at qualitative wall motion changes as an indirect marker for myocardial ischemia in the ACAOS subtended territories (71). However, the distal segments of the coronary arteries are not visible and therefore coronary dominance of the non-anomalous vs. anomalous vessel is not possible.

### Coronary Computed Tomography Angiography

With the substantial technical advances in the last decades, coronary computed tomography angiography (CCTA) has become the preferred imaging modality for anatomic definition of ACAOS in adults (5, 22). CCTA provides the best non-invasive spatial resolution and advanced post-processing methods as 3D virtual angiographic view enable the detailed evaluation of the anatomic high-risk features (28, 36, 45, 47, 74, 88–94). In addition and especially relevant in adult patients (34), CCTA allows the evaluation of the full course of the coronary arteries including detection of concomitant atherosclerotic CAD. In recent years, radiation exposure during CCTA has been dramatically reduced to an average of around 0.5–3 mSv in daily clinical practice (95). So far, CCTA was limited to the anatomical assessment of ACAOS. However, a novel technique may overcome this shortcoming by using computed fluid dynamic analysis, i.e., the implementation of CT fractional flow reserve (CT_FFR_) (96, 97) in patients with ACAOS (98). While first results are promising (98–101), CT_FFR_ has been primarily used in the evaluation of CAD and its diagnostic value in other setting remains unclear. To which degree CT perfusion (using dobutamine) may play a role in assessing ACAOS needs to be determined (102).

### Cardiac Magnetic Resonance Imaging

Cardiac magnetic resonance (CMR) imaging offers tomographic 3D imaging at high spatial resolution [slightly lower than CCTA (22, 54, 88)] without radiation at the expense of prolonged scan times and higher costs (5). It allows the visualization and assessment of the origin and the course of the ACAOS in relation to the great vessels in detail and without the use of contrast agents, rendering this modality especially attractive in the pediatric population (74, 103–105). CMR is able to capture additional relevant information related to cardiac structures and function (22), including myocardial necrosis as substrate for ventricular tachyarrhythmias (8). However, CMR is limited by its difficulty to assess the distal segments of the coronary arteries, as well as concomitant CAD. Concerning functional ischemia testing, CMR allows to investigate the hemodynamic relevance by pharmacologic inotropic stress (i.e., dobutamine) (106, 107) with a higher accuracy than stress echocardiography (108).

### Nuclear Cardiac Imaging

Nuclear cardiac imaging modalities [i.e., single-photon emission computed tomography (SPECT) and positron emission tomography (PET)] are established techniques for risk stratification and assessment of myocardial perfusion in the setting of CAD. Multiple studies used these modalities for the assessment of hemodynamic relevance of ACAOS (34, 75, 88, 109–111) and demonstrated favorable diagnostic performance. Furthermore, combination with CT allows the allocation to the corresponding vessel territory, a situation often altered in ACAOS (34, 109). However, as shown by a recent case report from our group (30), the limited spatial resolution may lead to undetected ischemia, in particular in cases with primary subendocardial ischemia.

### Invasive Coronary Angiography

Invasive coronary angiography (ICA) has been the gold standard for the diagnostic of CAAs for several decades. However, it is less suited to visualize anatomic high-risk features and to determine the ACAOS course in relation to the great vessels. Owing to the advent of non-invasive imaging modalities as CCTA and CMR, ICA is no longer a first-line modality to define the anatomy of ACAOS (75, 112). Nevertheless, in combination with intravascular diagnostic procedures such as intravascular ultrasound (IVUS) and optical coherence tomography (OCT), ICA continues to have clinical significance. According to Angelini et al., IVUS is the gold standard for the assessment of the intramural segment since it allows the best spatial assessment as well as the demonstration of dynamic lateral compression during simulated exercise (46, 54). Both, determination of the pressure gradient (i.e., FFR) across the anomalous segment as well as IVUS, are possible under simulation of physical exercise, allowing the most comprehensive evaluation of the hemodynamic relevance to date (27, 56–59, 66, 113, 114). Moreover, non-invasive functional testing does not allow to uncover possible isolated right ventricular ischemia (e.g., in R- ACAOS with a small RCA and left coronary dominance), as only the left myocardium can be assessed. Although, the myocardium at risk might be rather small in these situations, one could argue that arrhythmias still can be induced from the right ventricle and should be assessed using invasive FFR.

### Stress Testing

The ideal stress test for ACAOS should be able to assess both dynamic and fixed components, and has to be strenuous enough to provoke lateral compression. This requirement was illustratively demonstrated in a case report by Lim et al. (67), describing a 14-years old female patient with L-ACAOS that showed similar FFR_Adenosine_ and FFR_Dobutamine_ (0.87 vs. 0.86) values at a heart rate of 153 bpm (74% of the maximal heart rate) and thus, only evaluation of the fixed component. Hence, maximal exercise load is crucial and the examiners should aim for maximal or supramaximal stress (100% of predicted maximal heart rate or above, estimated with the formula of 220–age). Unfortunately, most performed stress tests were satisfied with 85% of the maximal heart rate (34, 56, 75, 111, 115), providing a possible explanation for the low reliability and the missing correlation with clinical symptoms and prognosis (46). Table 3 provides an overview of commonly used stress protocols and their application in non-invasive and invasive diagnostic modalities. In general, maximal physical exercise should be preferred. However, this is often not feasible, especially in the invasive setting. Further, pure vasodilators (i.e., adenosine or regadenosone) are not able to provoke the dynamic components (i.e., dynamic lateral compression of the intramural course) and thus, are prone to provide false negative results. In a small case series, the lateral compression illustrated by IVUS during ICA was provoked with norepinephrine (59). However, this method does not simulate vigorous physical exercise adequately because of only slightly increased heart rate and inadequate adaption of coronary vascular resistance (116).

**Table 3:** Overview of possible stress protocols in assessing patients with ACAOS.

	**Physical exercise**		**Adenosine**	**Regadenoson**	**Norepinephrine**	**Dobutamine**	**Dobutamine + volume challenge**
Protocol/dose	85% of max. HR	100% of max. HR	140 μg/kg/min	Bolus: 400 μg	0.01 μg/kg/min	40 μg/kg/min	40 μg/kg/min + saline: 1.5–3 l+ atropine: 1 mg
Applied in	Non-invasive testing	Non-invasive testing	Non-invasive / invasive testing	Non-invasive testing	Invasive testing	Non-invasive / invasive testing	Invasive testing
Increase in coronary blood flow to detect relevant fixed stenosis	+++	+++++	+++	+++	++	++++	++++
Increased heart minute volume to provoke dynamic lateral compression	++	+++++	-	-	+++	++	++++
Reproducibility of symptoms	+++	+++++	-	-	++	++	+++
Tolerability	++++	++++	++	+++	++	++	++

*HR, heart rate*.

For invasive stress testing, Angelini et al. introduced a “SAD”-test, that entails a pharmacologic stress test with rapid infusion of 500 ml saline, dobutamine stepwise infusion up to 40 μg/kg/min and in addition 0.5 mg atropine if the heart rate is below 140 bpm at the end of the dobutamine infusion (46, 54). While this stress protocol is the closest equivalent to vigorous exercise, it has two major limitations. First, a fixed target heart rate of 140 bpm lacks age-related adaption and thus, leads to insufficient exercise load among younger patients. Second, infusion of saline is necessary, since dobutamine decreases the preload and hence, systolic arterial blood pressure, aortic wall stress and myocardial oxygen consumption. However, as with the fixed heart rate, infusion of saline should have the extent to prevent blood pressure decrease during infusion of the dobutamine and maintaining an adequate preload rather than a fixed value.

Thus, our specialized clinic for ACAOS applies a more aggressive approach with steady infusion of saline during the whole invasive procedure (usually more than 1'500 ml of saline to prevent a preload decrease) and attempts to reach 100% of the maximal heart rate, i.e., using atropine in addition to the ongoing dobutamine infusion to simulate vigorous physical exercise at the upper limit. The dobutamine and volume challenge is, of course, not practicable for every patient but should be aimed for in order to simulate maximal physical exercise and obtain conclusive results even in absence of ischemia (i.e., true-negative results).

Regarding the invasive diagnostic procedure, radial access represents the preferred access site. The intubation of the anomalous ostium in combination with advanced diagnostic including FFR and intravascular imaging under rest and stress condition requires a high level of experience and should be reserved for experienced interventional cardiologists. Potential but rare risks include aortic or coronary dissections and stroke.

### Diagnostic Management of Patients With ACAOS

**Figure 3:**
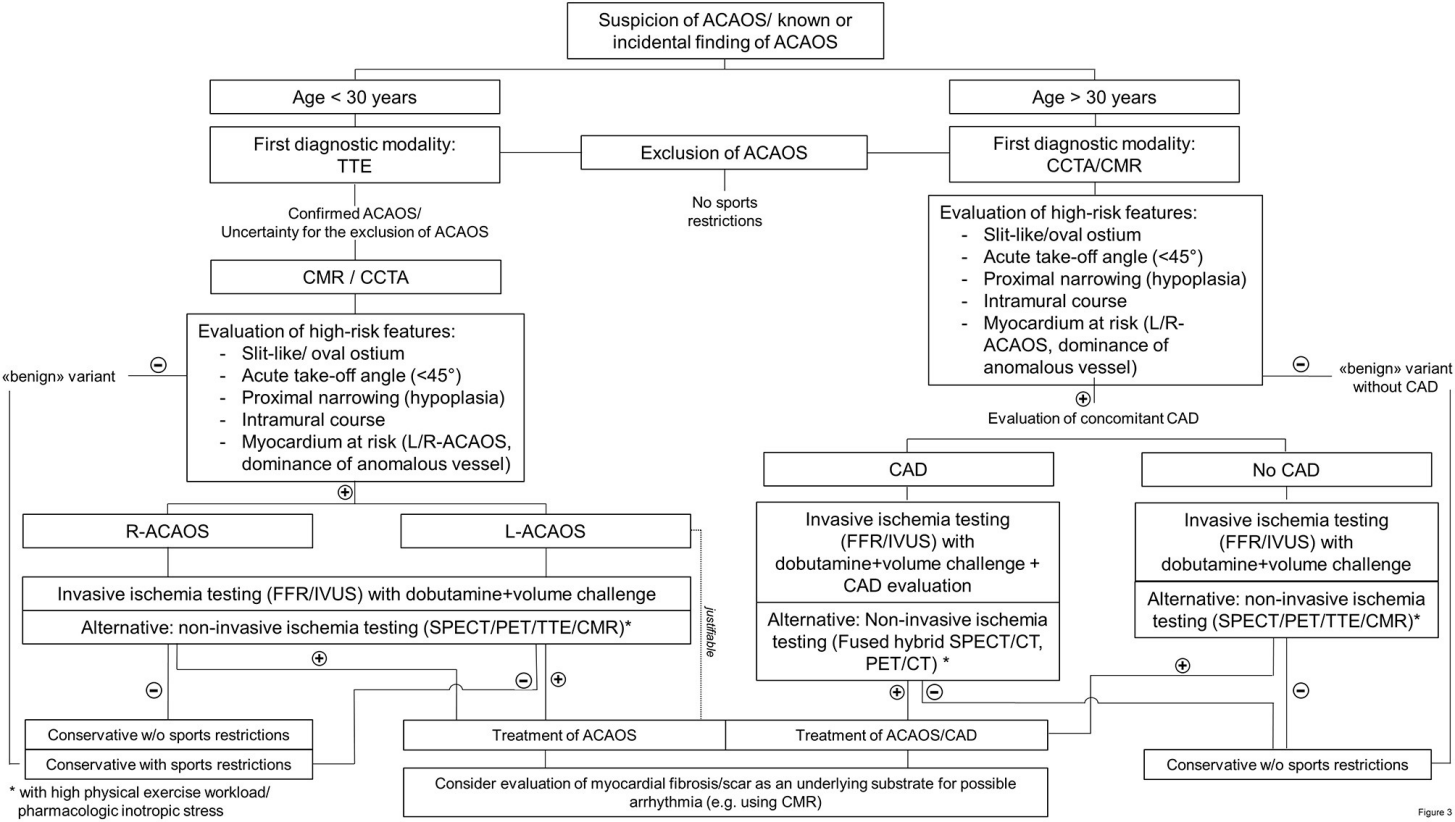
Flow chart of diagnostic management in patients with an anomalous coronary artery. (R-/L)-ACAOS, (right/left) anomalous coronary arteries with the origin of the anomalous vessel from the opposite sinus of Valsalva; CAD, coronary artery disease; CCTA, coronary computed tomography angiography; CMR, cardiac magnetic resonance imaging; FFR, fractional flow reserve; IVUS, intravascular ultrasound; PET, positron emission tomography; SPECT, single-photon emission computed tomography; TTE, transthoracic echocardiography.

After detailed recording of the medical history including symptoms, physical activities and strenuous exercise related symptoms, we propose the following downstream testing algorithm (i.e., summarized in a flow chart in Figure 3) in individuals with suspected or confirmed ACAOS. We divided the population into those below and above 30 years according to the studies by Taylor et al. (24, 38). We are fully aware that this dichotomization is arbitrary and should not be seen as a stringent recommendation but is rather meant for guidance.

#### Patients Under 30 Years of Age

In patients under 30 years of age (and especially in the pediatric population), the initial diagnostic modality should be TTE by an experienced sonographer. If ACAOS cannot be ruled out with certainty (because of inexperience, low acoustic window quality and/or others) or in cases where ACAOS is confirmed, additional imaging is required. For the subsequent diagnostic step, CCTA or CMR are the recommended diagnostic modalities, based on the local expertise and availability. Using these imaging methods, evaluation of anatomic high-risk features is crucial to directly rule out “benign” variants of CAA. Thus, ACAOS without any anatomic high-risk features can be safely deferred (5, 22), respectively referred to for further evaluations of the underlying causes in symptomatic patients.

Non-invasive functional testing is recommend when considering the association of cardiovascular events in ACAOS with strenuous exercise. This is, however, only useful when turning out positive or as reference value for subsequent evaluations. As stated by Cheezum et al., “the absence of ischemia during stress testing cannot be viewed as reassuring currently, particularly when potentially high-risk anatomic features are present” (5). In addition, we propose that every ACAOS with anatomic high-risk features should undergo an invasive evaluation of the hemodynamic relevance including assessment of IVUS and FFR under a dobutamine and volume challenge and non-invasive imaging should rather be seen as an alternative. If there is no evidence for ischemia and the patient remained asymptomatic, a conservative approach should be justifiable. In the other situations, revascularization should be recommend (14, 16) as well as a CMR (if not already done) for the evaluation of patchy myocardial necrosis.

#### Patients Over 30 Years of Age

In patients over 30 years of age, the diagnostic scheme is similar. However, in this setting, concomitant CAD must be ruled out. Accordingly, first-line modality is CCTA, followed by the same diagnostic procedures as outline above. Please note, even if TTE is not recommended as first-line modality in this population, we believe that it is an integral part of a cardiac diagnostic workup in adult people (similar to the ECG).

## Gaps of Knowledge

Multiple gaps of knowledge exists in ACAOS regarding the optimal diagnostic evaluation, risk stratification and management. As outlined by Brothers et al., we are not yet able to distinguish which individuals and which variants of ACAOS are at high risk for ischemia and who should we refer for revascularization (14).

The main questions are:

1. What is the prognosis of patients with only few/milder versions of anatomical high-risk features (e.g., short intramural course)? What are cut-off values for acute take-off angle, intramural length, height-width ratio of the slit like ostium that associated with an increased risk for adverse cardiac outcomes?
2. Does the decreased risk for SCD in newly detected ACAOS in older people represents a selection-bias toward a low-risk population (higher-risk individuals died at a younger age) or does the normal development in this patient cohorts based on pathophysiologic alternation (e.g., increased stiffness of the aortic wall) lead to a decreased lateral dynamic compression of the anomalous segment?
3. Does discrepancy between different invasive hemodynamic parameters [systolic/diastolic, resting and stress parameters (117)] represent valuable information on the aortic wall distensibility and the hemodynamic relevance?
4. Are sports restriction recommendations (dynamic vs. static sports, recreation vs. competitive sports) and revascularization necessary for all patients with high-risk features? What is the relevance of the age and the symptomatic burden on sports counseling?
5. Is it possible to predict the hemodynamic relevance of ACAOS purely based on non-invasive anatomical description of the high-risk features?

Ongoing studies:

Currently, several single site and multinational registries (118, 119) are recruiting patients to address the remaining gaps. Our site currently recruits patients for the systematic evaluation of ACAOS (NCT04475289) including non-invasive imaging (CCTA, stress-testing) as well as comprehensive invasive functional assessment. Our hypothesis is that the exact description of the anatomical features in the CCTA can determine the hemodynamic relevance of ACAOS using the invasively measured FFR_Dobutamine_ as reference.

## Conclusion

Despite numerous efforts to uncover the enigma of the hemodynamic relevance in patients with ACAOS, our understanding of the complex interactions leading to myocardial ischemia, remains unsatisfactory. Due to the low prevalence in the general population, major efforts have to be made to collect data from multinational ACAOS registries to better understand the pathophysiology of this entity. We advocate a two-tier concept, where the hemodynamic relevance of ACAOS is represented by a fixed component (e.g., proximal narrowing; similar to CAD) and a dynamic component (e.g., lateral compression), accentuated during exercise, providing explanations for the various clinical presentations. Hence, comprehensively assessment of the hemodynamic relevance of ACAOS should contain multimodality non-invasive and invasive imaging with adequate stress testing.

## Author Contributions

All authors listed have made a substantial, direct and intellectual contribution to the work, and approved it for publication.

## Conflict of Interest

LR received research grants to the institution by Abbott Vascular, Biotronik, Boston Scientific, Medis, Sanofi, and Regeneron and consultation/speaker fees by Abbott Vacular, Amgen, AstraZeneca, Canon, Occlutech, and Vifor. YU reports personal fees from Infraredex, outside the submitted work. CG received travel and conference fees from Amgen. SW reports research and educational grants to the institution from Abbott, Amgen, BMS, Bayer, Boston Scientific, Biotronik, Cardinal Health, CardioValve, CSL Behring, Daiichi Sankyo, Edwards Lifesciences, Johnson & Johnson, Medtronic, Querbet, Polares, Sanofi, Terumo, Sinomed and serves as unpaid member of the steering/excecutive group of trials funded by Abbott, Abiomed, Amgen, BMS, Boston Scientific, Biotronik, Cardiovalve, Edwards Lifesciences, MedAlliance, Medtronic, Polares, Sinomed, V-Wave and Xeltis, but has not received personal payments by any pharmaceutical company or device manufacturer. He is also member of the steering/excecutive committee group of several investigated-initiated trials that receive funding by industry without impact on his personal remuneration.

The remaining authors declare that the research was conducted in the absence of any commercial or financial relationships that could be construed as a potential conflict of interest.
